# Exhaled-Breath Testing Using an Electronic Nose during Spinal Cord Stimulation in Patients with Failed Back Surgery Syndrome: An Experimental Pilot Study

**DOI:** 10.3390/jcm10132921

**Published:** 2021-06-29

**Authors:** Lisa Goudman, Julie Jansen, Nieke Vets, Ann De Smedt, Maarten Moens

**Affiliations:** 1 Department of Neurosurgery, Universitair Ziekenhuis Brussel, 1090 Brussels, Belgium; Julie.Jansen@uzbrussel.be (J.J.); Nieke.Vets@uzbrussel.be (N.V.); Maarten.Moens@uzbrussel.be (M.M.); 2 STIMULUS Consortium (reSearch and TeachIng neuroModULation Uz bruSsel), Universitair Ziekenhuis Brussel, 1090 Brussels, Belgium; Ann.DeSmedt@uzbrussel.be; 3 Center for Neurosciences (C4N), Vrije Universiteit Brussel, 1090 Brussels, Belgium; 4 Pain in Motion International Research Group, 1090 Brussels, Belgium; 5 Department of Physical Medicine and Rehabilitation, Universitair Ziekenhuis Brussel, 1090 Brussels, Belgium; 6 Department of Radiology, Universitair Ziekenhuis Brussel, 1090 Brussels, Belgium

**Keywords:** volatile organic compounds, breath tests, electronic nose, neuromodulation, chronic pain

## Abstract

The increased awareness of discrepancies between self-reporting outcome measurements and objective outcome measurements within the field of neuromodulation has accelerated the search towards more objective measurements. The aim of this study was to evaluate whether an electronic nose can differentiate between chronic pain patients in whom Spinal Cord Stimulation (SCS) was activated versus deactivated. Twenty-seven patients with Failed Back Surgery Syndrome (FBSS) participated in this prospective pilot study. Volatile organic compounds in exhaled breath were measured with electronic nose technology (Aeonose™) during SCS on and off states. Random forest was used with a leave-10%-out cross-validation method to determine accuracy of discriminating between SCS on and off states. Our random forest showed an accuracy of 0.56, with an area under the curve of 0.62, a sensitivity of 62% (95% CI: 41–79%) and a specificity of 50% (95% CI: 30–70%). Pain intensity scores were significantly different between both SCS states. Our findings indicate that we cannot discriminate between SCS off and on states based on exhaled breath with the Aeonose™ in patients with FBSS. In clinical practice, these findings imply that with a noninvasive electronic nose, exhaled breath cannot be used as an additional marker of the effect of neuromodulation.

## 1. Introduction

One of the treatment options for a variety of chronic pain conditions is Spinal cord stimulation (SCS), a minimally invasive neuromodulation technique [1]. SCS is a well-known treatment option for patients with Failed Back Surgery Syndrome (FBSS) who are refractory to conventional management, with positive effects on the pain experience and quality of life [1,2,3,4,5]. The exact mechanisms of SCS are not yet fully elucidated; however, several working mechanisms and hypotheses have been proposed on spinal, segmental and supraspinal level [6,7]. Additionally, during the latest years it is assumed that SCS induces alterations in the autonomic nervous system with an upregulation of the parasympathetic system, based on heart rate variability, respiration, and skin conductance measurements [8,9]. In the field of neuromodulation, and in chronic pain trials in general, patient-reported outcomes are still the primary choice to serve as outcome measurements [10]. Within pain intensity reporting, patients are often asked to provide a quantitative rating for their pain experience by converting a subjective feeling into a quantitative number [11]. Problems occur when patients experience extremely high pain or minimal pain, compared to other patients, which is related to the problem of how a third-person observer can appreciate the first-person experience of another individual [12]. To facilitate communication, there is a need for objectively measuring output mechanisms of the individual experience of pain [13], complementary to the personal patient reporting.

One of the latest non-invasive technologies that has gained attention during the last decade is the use of electronic noses to measure exhaled breath and more specifically volatile organic compounds (VOCs), which could be used a more objective tool to evaluate the effects of output mechanisms of pain and neuromodulation. Electronic noses are preferred above gas chromatography or mass spectrometry since the latter are complex, costly and time-consuming [14], compared to the straightforward application of electronic noses [15]. Nevertheless, gas chromatography-mass spectrometry is still considered the gold standard for identification of VOCs due to its specificity in identifying the actual presence of a particular substance in a given sample [16,17]. The composition of exhaled breath is a mixture of nitrogen, oxygen, carbon dioxide, water, and inert gases [18]. The remaining small fraction of human breath consists of trace components, of which more than 500 have been described [18]. These VOCs, among which ethane, pentane, isoprene, or acetone, are mainly blood borne and thus enable monitoring of different processes in the body. Additionally, they can provide insights into different biochemical processes in the healthy and the diseased human body [18] among which applications to predict lung cancer [19], distinguish inflammatory bowel disease from healthy controls, and ulcerative colitis from Crohn’s disease [20].

Since exhaled breath contains hundreds of different VOCs, each individual has a unique volatile chemical breath print [21]. Electronic noses are composites of a sensor array and an in-built processor [22] whereby the sensor array gives a signal pattern after activation by an odour (i.e., they are not selective for individual molecules) [23]. Thus, after measuring the exhaled volatile compound sample of breath, the main principle behind an electronic nose is to apply pattern recognition techniques to complex measurement data [23,24]. In order to identify specific scents that are present within a complex mixture, the measured response is compared to a previously observed response (i.e., pattern recognition) [24]. Except for one study in which the volatile organic profiles of patients with complex regional pain syndrome were successfully discriminated from the profiles of healthy controls [25], no specific response pattern within the field of chronic pain has yet been identified. The aim of this pilot study is to measure VOCs in exhaled breath in patients with Failed Back Surgery Syndrome (FBSS) with an electronic nose, to discriminate between on and off states of Spinal Cord Stimulation (SCS).

## 2. Materials and Methods

### 2.1. Participants

In this study, patients with FBSS who received treatment with SCS at the department of Neurosurgery of Universitair Ziekenhuis Brussel were invited to participate. Every six months, according to routine clinical care at this center (and regulated by reimbursement rules), all patients have a standard clinical visit regarding their treatment progress. Those patients who were scheduled for a 6-month SCS follow-up visit, were invited to take part. Only patients with a minimum age of 18 years were allowed to participate. Patients were not allowed to take part in the study if they were previously diagnosed with major psychiatric problems, previously received a diagnosis of cancer or if they have an underlying respiratory disease.

The study protocol was approved by the central ethics committee of Universitair Ziekenhuis Brussel (B.U.N. 1432020000074) on 27 May 2020. The study was registered on clinicaltrials.gov (NCT04469738) on 14 July 2020. All patients provided written informed consent before participation. The study was conducted according to the revised Declaration of Helsinki (1998).

### 2.2. Protocol

This is a pilot study with an experimental design, consisting of a single outpatient visit. All patients who took part in this study were instructed to switch off SCS 12 h before their study visit [11]. During the study visit, exhaled breath was first measured when SCS was still switched off. Afterwards, patients were asked to provide a pain intensity score. After this measurement, patients had to switch their neurostimulator on, followed by a rest period of at least 30 min. A second evaluation of exhaled breath and pain intensity reporting was conducted after this 30 min break. Due to the pain-relieving effects of SCS, patients could not be blinded to study condition.

All patients were asked to confirm that they switched off SCS 12 h before the study visit. This statement was controlled by evaluating whether SCS was effectively switched off (which was the case for all patients) when patients presented themselves for the study visit. No specific instructions regarding medication use where provided, so patients could continue with their current medication on the day of the measurements.

### 2.3. Aeonose™

The Aeonose™ (The eNose Company, Zutphen, The Netherlands) is a handheld, battery-powered electronic nose which enables to analyse volatile organic compounds. When breathing through the device, exhaled breath is guided over three small hotplate metal-oxide sensors, which behave as semi-conductors at higher temperatures [26]. The metal-oxide sensors are periodically heated in cycles of 20 s using a 32-step sinusoidal modulation of the sensor surface temperature [27]. A broad range of VOCs in the exhaled breath induce a redox reaction on those sensor surfaces, depending on temperature oscillations, which lead to a change in the conductivity that can be measured and quantified, resulting in a unique breath signal [24]. A full measurement takes 15 min, of which the patient is breathing though the Aeonose™ for 5 min and 10 min are used for desorption, cleaning and recovery [27]. A Bluetooth connection was used to transfer data from the Aeonose™ to the central server for data analysis, hosted by eNose Company.

Patients were instructed to inhale and exhale through a disposable mouthpiece of the Aeonose™ for 5 consecutive minutes. To ensure all air circulation went through the Aeonose™, patients received a nose clamp (to exclude nose breathing) and were instructed to enclose their lips over the mouthpiece at all times. Additionally, to eliminate exogenous influences on breath patterns, the mouthpiece contained a carbon filter to ensure the air was filtered and a HEPA filter to prevent contamination of the internal tubing of the device [26]. To further eliminate possible exogenous VOCs, the first two minutes of each 5 min measurement were only used to rinse the air in the lungs. The remaining three minutes were used in the analysis [21]. To familiarise patients with the device, patients could perform a few in- and exhales to get acquainted with the Aeonose™ before the actual measurements started. All measurements with the Aeonose™ took place in the same room. No alcohol gel, nor hand sanitizers were allowed in the room where the measurements took place. Both the researcher and patients wore gloves all the time during the experiment.

### 2.4. Self-Reported Outcome Measurements

The Visual Analogue Scale (VAS) was used to assess current pain intensity for low back pain and leg pain separately. Therefore, a 10 cm line was provided to all patients in paper format, representing a continuum between no pain and maximal pain. Pain intensity is expressed in mm on a scale from 0 to 100. Patients completed this questionnaire twice; once when SCS was switched off for 12 h and once when SCS was reactivated for 30 min. The VAS pain score is a reliable and valid tool that is sensitive to change [28,29,30,31].

The Medication Quantification Scale III (MQS) was used to quantify medication use [32]. For each medication, a MQS score is calculated by multiplying a detriment weight for a given pharmacologic class with a score for dosage [33]. Five different classes of medication are described within the MQS: nonsteroidal anti-inflammatory drugs (NSAIDs) and acetaminophen, muscle relaxants, neuropathic pain medications (antidepressants and anticonvulsants), benzodiazepines, and opioids. All calculated values are summed to obtain a total MQS score, whereby a score of zero indicates no medication use. The higher the total score on the MQS, the higher the negative impact of medication [33].

### 2.5. Sample Size Calculation

Due to the lack of previous studies in this population, an exact sample size calculation for this pilot study was deemed unfeasible. For a standard proof-of-concept study with an electronic nose, 25 patients are required [21,34]. Therefore, in this pilot study we aimed to include at least 25 patients with FBSS who are treated with SCS.

### 2.6. Statistical Analysis

All descriptive analyses were performed in R Studio version 1.4.1106 (R version 4.0, Vienna, Austria). *p*-values of 0.05 or less were considered statistically significant. Descriptive statistics are provided as mean (± SD) or as median (first and third quartile). A simple regression model was built with pain intensity scores as outcome variable and the on and off state of SCS and pain location (low back or leg) as explanatory variables. An automatic step function was applied to withheld relevant predictors.

Data analysis of exhaled-breath patterns was performed by Aethena, a proprietary software program (eNose Company, Zutphen, The Netherlands) which performs data compression, data classification and data reporting based on the raw data. The goal was to build a classifier to discriminate between both SCS groups (activated or deactivated). First, feature extraction was performed for data compression. Then, generated vectors were normalized and entered into a random forest (400 trees, minimum split size 2, maximum tree depth 5, minimum information gain 0.0010). Finally, leave-10%-out cross-validation was applied to determine the performance of the created model.

## 3. Results

### 3.1. Descriptive Statistics

In total, 27 patients were included in this study. All experiments were conducted between 12 December 2020 and 27 March 2021. One patient had a vagal syncope during the experimental measurement when SCS was switched off, wherefore the experiment was immediately terminated for this patient. Data of this patient were not included in the analysis, leading to a final dataset of 26 patients. Patients had a mean age of 56 (SD: 10) years and a median BMI of 27 (Q1–Q3:26–30) kg/m^2^. Twelve females (46%) and 14 males (54%) were included. The median duration that patients were implanted with SCS was 5.5 (Q1–Q3:2–8) years. The median score on the MQS was 9.15 (Q1–Q3:6.22–16.65). Patient characteristics are provided in Supplementary Table S1.

**Figure 1:**
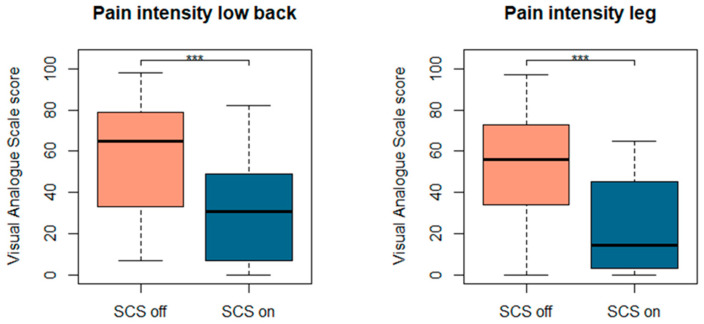
Pain intensity scores for lower back (**left**) and leg (**right**) during SCS off and SCS on conditions. Abbreviations. SCS: spinal cord stimulation, *** is denoting a statistically significant difference in pain intensity scores between SCS on and off states.

Pain intensity scores were significantly different between both SCS states with median values of 65 (Q1–Q3:36–78.50) and 56 (Q1–Q3:34–72.25) during SCS off states and 30.50 (Q1–Q3:7.50–48.25) and 14.50 (Q1–Q3:3.25–43) during SCS on states for low back and leg pain, respectively. A simple regression model for pain intensity scores revealed a significant effect of SCS condition (type III test: F = 30.23, *p* < 0.001) on pain intensity. Pain location (low back or leg) was not withheld in the final model (type III test: F = 1.58, *p* = 0.21). Figure 1 is presenting pain intensity scores for low back and leg pain separately.

### 3.2. Exhaled Breath

**Figure 2:**
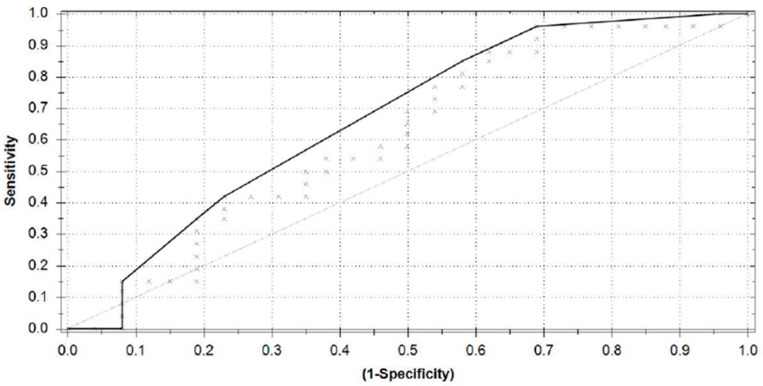
ROC curve for classifying patients with FBSS in SCS on or SCS off states.

Model performance is presented in Table 1. The model has an accuracy of 56% with a sensitivity of 62% (95% CI from 41% to 79%) and a specificity of 50% (95% CI from 30% to 70%). The area under the curve equals 0.62 (Figure 2).

Sensitivity is calculated as true positives/(true positives + false negatives), specificity as true negatives/(false positives + true negatives), positive predictive value as true positives/(true positives + false positives) and negative predictive value as true negatives/(false negatives + true negatives). Abbreviations. NPV: negative predictive value, PPV: positive predictive value, SCS: spinal cord stimulation, sens: sensitivity, spec: specificity.

## 4. Discussion

In this study we evaluated whether an electronic nose, by means of exhaled volatile organic compounds analysis, can discriminate between SCS on (activated for 30 min) and SCS off (deactivated for 12 h) states in patients with FBSS. Modern electronic nose technology is not focusing on individual compounds but creates a pathology-dependent signature, reflecting overall exhaled volatile organic compound content. For several pathologies, a specific nose (software algorithm) is developed that is able to diagnose certain diseases compared to a group without the disease, for example noses to diagnose tuberculosis [35], breast cancer [36] or differentiate asthma with fixed airways obstruction and chronic obstructive pulmonary disease [37] are already developed. For the differentiation between SCS activation and deactivation in chronic pain patients, no specific algorithm was available yet wherefore this pilot study was conducted to develop a signature of volatile organic compound content specifically for this population. Based on the results of this study, the constructed algorithm did not reveal a proper performance, wherefore we need to conclude that with exhaled breath analysis we cannot discriminate between both groups.

One of the potential explanations of this negative trial can be found in the lack of knowledge about wash-in periods of SCS in general and more specifically on the respiratory system. Up till now, the exact wash-in period of SCS is not yet unravelled, wherefore a time period of 30 min (in line with literature [9,38]) was used in this experiment to ensure SCS is functioning properly. Pain intensity scores significantly decreased during this 30 min break, indicating that a wash-in period of 30 min is sufficient for pain relief. Additionally, a previous study in this population already indicated that heart rate and respiration rate are altered within a time frame of 30 min after activating SCS [9]. Similarly, heart rate variability changes were previously revealed within this time frame, indicating an alteration of the parasympathetic nervous system after activating SCS [8]. Nevertheless, it is unclear whether the content of exhaled volatile organic compounds itself is substantially altered after this time period. A longitudinal cohort study in which patients are evaluated before SCS implantation and re-evaluated after final SCS implantation should be able to discard the issues related to wash-in periods. Moreover, the current research question was rather ambitious with trying to develop a nose for discriminating between pain intensity during SCS activation and deactivation. It might be possible that we first needed to develop a nose to differentiate between healthy controls and patients with FBSS, which can then be used to discriminate between pain intensity reporting in chronic pain patients. Bijl et al. (2019) previously compared exhaled breath of healthy controls and chronic pain patients, and more specifically patients with CRPS. They obtained an overall accuracy of 81% to discriminate between patients with CRPS and healthy controls based on analysis with the Aeonose™, indicating that a differentiation between the absence or presence of pain is possible with this measurement device [25]. Nevertheless, in our study, no differentiation was possible between treatment effects in a chronic pain population. Perhaps, only a discrimination between the absence or presence of pain is possible, without a further finetuning of the amount of pain. Finally, it might also be possible that exhaled breath is not altered by neuromodulation (i.e., absence of changes in VOCs) or that the device is not sensitive enough to detect small changes in VOCs, since a study with mass spectrometry revealed clear differences is several specific VOCs in patients with postoperative pain, before and after treatment with opioid analgesics [39], pointing at the potential to differentiate between pain states with mass spectrometry.

This is the first study exploring the use of an electronic nose in the context of neuromodulation. A literature review concluded that in order to better understand the complete mechanisms of action of SCS, it is necessary to carry out properly controlled experiments with objective outcome measures [40]. Several outcome measurements that are not relying on self-reporting of patients such as accelerometry, sweat gland function, heart rate variability have already been explored [8,9,41,42]. Additionally, more authors are striving towards a combination of self-reported and more objective outcome measurements in neuromodulation [43]. Nevertheless, the sensitivity and specificity of the Aeonose™ are not sufficient to implement this measurement tool in clinical practice to evaluate the effect of neuromodulation.

In this study, several limitations should be taken into account. We first need to acknowledge that patients were not instructed to omit the use of analgesics due to ethical reasons which could have influenced the results. It is suggested that medication could influence the concentration of volatile metabolites [23]. A study by Meka et al. (2007) suggested that medication compliance could be monitored by electronic noses [44]. Nevertheless, more studies are needed to fully understand the interplay between VOCs and medication. Additionally, for one patient, it was impossible to complete the 5 min measurement due to a vagal syncope during the experiment. Nevertheless, the Aeonose™ was feasible (95% successful data collection) to use and appeared to be a safe measurement instrument; patients were still able to perform the breathing experiment even in the presence of pain (SCS switched off). The HEPA and carbon filters on the Aeonose™ create an inhalation resistance, which can be problematic for patients. Finally, no formal sample size calculation was performed due to the lack of a known effect size in this population. Based on recommendations from the eNose Company, a minimum sample size of 25 patients is needed to properly train a model.

## 5. Conclusions

Our findings indicated that discriminating between SCS off and on states in patients with FBSS was not possible with exhaled breath, measured with Aeonose™.

## Figures and Tables

**Table 1:** Model performance of the random forest on the leave-10%-out cross-validation dataset.

		Actual Observation		
		SCS on	SCS off	
Model prediction	SCS on	16	13	PPV = 0.55
	SCS off	10	13	NPV = 0.57
		Sens = 0.62	Spec = 0.50	Total = 52

## Data Availability

Not applicable.

## Supplementary Materials

The following are available online at https://www.mdpi.com/article/10.3390/jcm10132921/s1, Table S1: Patient characteristics.
