# Supplementary material for: Exhaled-Breath Testing Using an Electronic Nose during Spinal Cord Stimulation in Patients with Failed Back Surgery Syndrome: An Experimental Pilot Study
Source: J Clin Med. 2021 Jun 29;10(13):2921. doi: 10.3390/jcm10132921 (PMC8269089; doi:10.3390/jcm10132921)
Supplement: Supplementary file 1 [file jcm-10-02921-s001.zip › jcm-1258344-supplementary.pdf]

Supplementary Materials

Supplementary Table S1. Patient characteristics.

**Sex**	**Age**	**Type of SCS**	**Surgical indication**	**MQS total score**	**MQS NSAID+acetaminophen subscore**	**MQS relaxant subscore**	**MQS neuropahtic mood agents subscores**	**MQS benzodiazepines subscore**	**MQS opioids subscore**	**Comorbidities**
M	59	conv	lumbar disc herniation	3.9	0	0	0	3.9	0	cardiovascular
M	67	conv	lumbar disc herniation	5.8	0	0	1.9	3.9	0	infection
M	55	conv	spinal tumor resection	17.3	0	0	13.9	0	3.4	
F	53	conv	lumbar disc herniation	0	0	0	0	0	0	
F	67	HD	lumbar disc herniation	15.3	0	0	8.5	0	6.8	fibromyalgia
M	42	conv	lumbar fusion	11	0	0	7.6	0	3.4	cardiovascular
F	75	conv	spinal decompression	6.8	6.8	0	0	0	0	

F	60	conv	spinal tumor resection	17	3.4	0	0	0	13.6	
M	44	conv	cervical disc herniation	11.6	0	0	8.2	0	3.4	
F	70	conv	lumbar disc herniation	6.2	3.4	0	2.8	0	0	cardiovascular
M	48	conv	lumbar fusion	14.1	0	0	0	3.9	10.2	
M	42	conv	lumbar fusion	18.9	0	0	1.9	0	17	
F	53	conv	lumbar disc herniation	32.2	3.4	0	4	7.8	17	DM
F	56	conv	spinal decompression	6.3	0	0	6.3	0	0	DM
M	51	conv	lumbar fusion + corpectomy	6.3	0	0	6.3	0	0	DM
F	55	conv	lumbar fusion	3.4	0	0	0	0	3.4	
M	36	HF-10	lumbar fusion	17	0	0	0	0	17	
M	53	HF-10	lumbar fusion	6.2	0	0	2.3	3.9	0	
F	49	HF-10	lumbar fusion	13.6	3.4	0	0	0	10.2	
M	62	HF-10	spinal decompression	7.3	0	0	0	3.9	3.4	sleep disorder
M	50	conv	lumbar fusion	15.6	0	0	0	15.6	0	

M	73	conv	lumbar disc herniation	6.8	6.8	0	0	0	0	cardiovascular
F	51	DTM	lumbar disc herniation	7.3	3.4	0	0	3.9	0	
M	61	conv	lumbar fusion	3.4	0	0	0	0	3.4	DM
F	72	conv	lumbar fusion	17.4	0	0	2.3	11.7	3.4	
F	46	conv	lumbar disc herniation	27.5	13.6	0	13.9	0	0	

Abbreviations. Conv: standard spinal cord stimulation, DM: diabetes mellitus, DTM : differential target multiplexed spinal cord stimulation, F: female, HD : high-dose spinal cord stimulation, HF-10 : high frequency spinal cord stimulation at 10 kHz, ID: patient identification, M: male, MQS: medication quantification scale, P: patient.
